# TIPE2 regulates periodontal inflammation by inhibiting NF-κB p65 phosphorylation

**DOI:** 10.1590/1678-7757-2023-0162

**Published:** 2023-07-24

**Authors:** Yanmei DU, Xiaohua LIU, Changjie XIAO, Jianbin LI, Zhenxian SHENG, Yuxin WANG, Ronglin WANG, Xijiao YU

**Affiliations:** 1 Jinan Stamotological Hospital Jinan Key Laboratory of Oral Tissue Regeneration Shandong Provincial Health Commission Key Laboratory of Oral Diseases and Tissue Regeneration Shandong Province China Jinan Stamotological Hospital, Jinan Key Laboratory of Oral Tissue Regeneration, Shandong Provincial Health Commission Key Laboratory of Oral Diseases and Tissue Regeneration, Shandong Province, China.; 2 Jinan Stamotological Hospital Jinan Key Laboratory of Oral Tissue Regeneration Central Laboratory Shandong Province China Jinan Stamotological Hospital, Jinan Key Laboratory of Oral Tissue Regeneration, Central Laboratory, Department of Endodontics, Shandong Provincial Health Commission Key Laboratory of Oral Diseases and Tissue Regeneration, Shandong Province, China.; 3 Jinan Stamotological Hospital Department of Prosthodontics Shandong Province China Jinan Stamotological Hospital, Department of Prosthodontics, Shandong Province, China.; 4 Binzhou Medical College School of Stomatology Shandong China Binzhou Medical College, School of Stomatology, Shandong, China.

**Keywords:** Tumor necrosis factor-α-induced protein 8-like 2, Porphyromonas gingivalis, Periodontitis, NF-κB, Inflammation

## Abstract

The roles and molecular mechanisms of tumor necrosis factor-α-induced protein 8-like 2 (TIPE2) in periodontitis remain largely unknown.

**Objective:**

This study aimed to determine the expression of TIPE2 and NF-κB p65 in rat Porphyromonas gingivalis-induced periodontics in vivo.

**Methodology:**

Periodontal inflammation and alveolar bone resorption were analyzed using western blotting, micro-computed tomography, TRAP staining, immunohistochemistry, and immunofluorescence. THP-1 monocytes were stimulated using 1 μg/ml Pg. lipopolysaccharide (Pg.LPS) to determine the expression of TIPE2 *in vitro*. TIPE2 mRNA was suppressed by siRNA transfection, and the transfection efficiency was proven using western blotting and real-time PCR. The NF-κB pathway was activated by treating the cells with 1 μg/ml Pg.LPS to explore related mechanisms.

**Results:**

The expression of both TIPE2 and NF-κB p65 was increased in the gingival tissues of rat periodontitis compared with normal tissues. Positive expression of TIPE2 was distributed in inflammatory infiltrating cells and osteoclasts in the marginal lacunae of the alveolar bone. However, strong positive expression of TIPE2 in THP-1 was downregulated after Pg.LPS stimulation. TIPE2 levels negatively correlated with TNF-α and IL-1β. Decreased TIPE2 in THP-1 further promoted NF-κB p65 phosphorylation. Mechanistically, TIPE2 knockdown upregulated NF-κB signaling pathway activity.

**Conclusions:**

Taken together, these findings demonstrate that TIPE2 knockdown aggravates periodontal inflammatory infiltration via NF-κB pathway. Interventions aimed at increasing TIPE2 may help in the therapeutic applications for periodontitis.

## Introduction

Periodontitis is one of the most common human inflammatory diseases, which is closely related to both bacterial infections and host immunity.^1,2^ A dysregulated immune response can lead to aggravated periodontal inflammation and bone resorption by allowing the excessive recruitment of pro-inflammatory cells to the periodontal tissues.^3^ Perturbed immune cells, including monocytes/macrophages in periodontitis-affected sites, play a critical role in the pathogenesis of periodontitis.^4,5^ Studies have shown that both negative regulation of inflammatory cells and interventions aimed at increasing anti-inflammatory cells can assist in therapeutic applications for periodontitis.^6^ Therefore, it is of interest to explore effective molecular targets for immune cell regulation.

Tumor necrosis factor-α-induced protein 8-like 2 (TIPE2), a member of the tumor necrosis factor-alpha-induced protein-8 family, is a novel negative regulator of innate and adaptive immune responses, and its selective immune expression maintains immune homeostasis and prevents hyperresponsiveness. TIPE2 is preferentially expressed in lymphoid tissues, and its deletion leads to lethal Toll-like receptor and T cell receptor activation, multiorgan inflammation, splenomegaly, and premature death.^7^ TIPE2 inhibits M1 macrophage-related neutrophilic inflammation in asthma^8^ and accelerates IL-4-induced immunomodulatory M2 differentiation.^9^ Decreased TIPE2 in peripheral blood mononuclear cells is associated with the disease progression of chronic hepatitis,^10^ asthma,^11^ systemic lupus erythematosus,^12^ and osteoporosis.^13^ TIPE2 protects cardiomyocytes from ischemia-reperfusion-induced apoptosis by decreasing cell autophagy via the mTORC1 signaling pathway.^14^

Reduced TIPE2 expression is inversely associated with proinflammatory cytokines and positively correlated with bone mineral density.^13^ TIPE2 has also been shown to promote host resistance to *Pseudomonas aeruginosa* and inhibit NF-κB signaling and inflammatory infiltration in keratitis.^15^

Although studies have highlighted that TIPE2 is an important regulator of the immune response in various diseases, the roles and molecular mechanisms of TIPE2 in periodontitis remain largely unknown.

In this study, we investigated the expression of TIPE2 in rat periodontitis induced by *Porphyromonas gingivalis in vivo*. Additionally, we stimulated THP-1 monocytes with 1 μg/ml of Pg. lipopolysaccharide (Pg.LPS) to determine the expression of TIPE2 *in vitro*. Targeting TIPE2 in the immune response may prove to be beneficial in facilitating the healing process of periodontitis.

## Methodology

### Animals

A total of 18 cage-reared male Wistar rats (6-week-old, weight: 260–280 g) were provided with adequate normal hard food and water under a suitable laboratory feeding environment (12-h light/12-h dark cycle, 22–24°C, and 45% relative humidity). *Porphyromonas gingivalis* lipopolysaccharide (Pg.LPS) (InvivoGen, Hong Kong) was inoculated into a 4–0 silk suture, which was tied firmly around the dental neck of the bilateral maxillary second molars of 12 rats for two and four weeks. The use of rats conformed to the regulations of the Ethics Committee of Jinan Stomatological Hospital (No. JNSKQYY-2021-009). Gingival tissues from both sides of the maxillary second molars were taken from six rats for western blotting two weeks after ligation, whereas gingival tissues from six normal rats were used as controls (n=6). Additionally, six left maxillary bones from six rats were collected four weeks after ligation for micro-computed tomography (CT), whereas six right maxillary bones were taken for TRAP staining, immunohistochemistry, and immunofluorescence.

### Micro-CT scan

A micro-CT system (SkyScan 1176, Belgium) was used to observe alveolar bone resorption of the maxillary second molars. The scanning parameter was set at 65 kV/380 µA.

### TRAP staining

A commercial kit (Joytech Bio. Co., Zhejiang, China) was used for TRAP staining. Osteoclasts (OCs) containing three or more nuclei were identified as TRAP-positive multinucleated cells.

### Immunohistochemical staining and immunofluorescence

A Streptavidin-Peroxidase kit (Zhongshan, Beijing, China) was used strictly following the manufacturer’s instructions. The polyclonal antibody TIPE2 (1:100, Proteintech, USA) was used for immunohistochemical staining. Fluorescein and rhodamine (TRITC)-conjugated goat anti-rabbit IgG (secondary antibody) was used for immunofluorescence, with phosphate-buffered saline used as a negative control.

### Western blotting analysis

The MinuteTM Total Protein Extraction kit (Inventbiotech, Plymouth, MN, USA) was used to extract total proteins. Briefly, 10% SDS-PAGE was used to separate equal proteins, which were subsequently electroblotted to polyvinylidene difluoride (PVDF) membranes (Millipore, Billerica, MA, USA). After blocking with 5% nonfat milk, the primary antibodies, including TIPE2 (1:500, Proteintech, China), p38, p65, p p65 (1:1000; Cell Signaling Technology), and β-actin (1:500, Servicebio), were added and incubated overnight at 4°C. Horseradish peroxidase-goat anti-rabbit IgG (1:10000; CWBiotech, Beijing, China) was incubated at room temperature for 1 h. Finally, the blots were visualized with an Amersham Imager 800 instrument.

### Cell culture

The THP-1 human leukemia monocytic cell line was purchased from Haixing Biosciences and cultured in a special culture medium (TCH-G361, Haixing Biosciences, China). THP-1 cells were stimulated using 1 μg/ml Pg.LPS for 24 h to detect the expression of TIPE2. Subsequently, the THP-1 cells were transfected with siRNA for 48 h, with the empty body group used as the control. Next, the THP-1 cells were stimulated with 1 μg/ml Pg.LPS for 0, 10, and 20 min to detect NF-κB p65 phosphorylation.

### Small interference RNA (siRNA)

THP-1 cells (1×10^5^ cells/well) were plated on six-well plates and cultivated to an 80% degree of fusion. Dry powder siRNA was added to the solution and prepared into a working fluid of 20 μM. Next, 1.75 ml of complete medium and 250 μl of transfection solution were added per well to verify the efficiency of siRNA. The sequences of TIPE2 siRNA were as follows: sense, 5’-GCAGUGAGGUGCUAGAUGATT-3’; antisense, 5’-UCAUCUAGCACCUCACUGCTT-3’. The cells were transfected and cultured in an incubator at 37°C and 5% CO2 for 48 h, before conducting qPCR and western blot to verify the knockdown effect.

### Enzyme-linked immunosorbent assay (ELISA)

Cell culture supernatants were collected to detect the levels of IL-1β and TNF-α using ELISA (Multiscience, Hangzhou, China) following the manufacturer’s instructions.

### Real-time PCR (qPCR)

**Figure 1:**
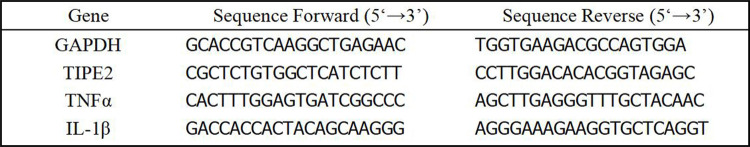
Primers used in real-Time PCR

Cellular total RNA was extracted using FastPure^®^ Cell/Tissue Total RNA Isolation Kit V2 (Vazyme Biotech, China) and used to synthesize cDNA with HiScript^®^III RT SuperMix for qPCR. Real-time PCR (20 μl total volume) was accomplished using ChamQ Universal SYBR qPCR Master Mix (Vazyme Biotech, China). GAPDH was used as a standardized reference. The results were presented indirectly using the 2−ΔΔCT method. The primers used are shown in Figure 1.

### Statistical analysis

All data are presented as mean ± standard deviation. The data were analyzed using non-parametric tests using GraphPad Prism 8.0 software (San Diego, CA, USA). Statistical significance was defined as p<0.05.

## Results

### Increased TIPE2 in gingival tissues of rat periodontitis

According to western blot results, the expression of NF-kB p65 and p38 was significantly increased in the gingival tissues, suggesting that NF-kB and p38 pathways were activated and that gingival inflammation occurred.

**Figure 2:**
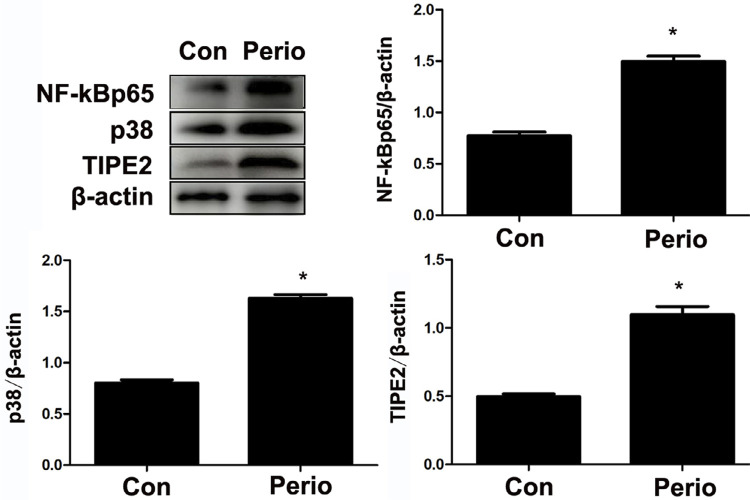
Two weeks after ligation, the gingival tissues from the rat’s second maxillary molar were extracted. The western blot results showed that the expression of NF-kB p65 and p38 was significantly increased, suggesting that the classical inflammatory NF-kB and p38 signaling pathways were activated, and gingival inflammation occurred. The expression of TIPE2 in the inflammatory gingival tissues was also significantly higher than that in the normal gingival tissues, suggesting that TIPE2 participates in periodontal inflammation. *p<0.05

The expression of TIPE2 in the inflammatory gingival tissues was also significantly higher than that in normal gingival tissues, suggesting that TIPE2 participated in gingival inflammation (Figure 2).

### Histochemical localization of TIPE2 in rat periodontitis

**Figure 3:**
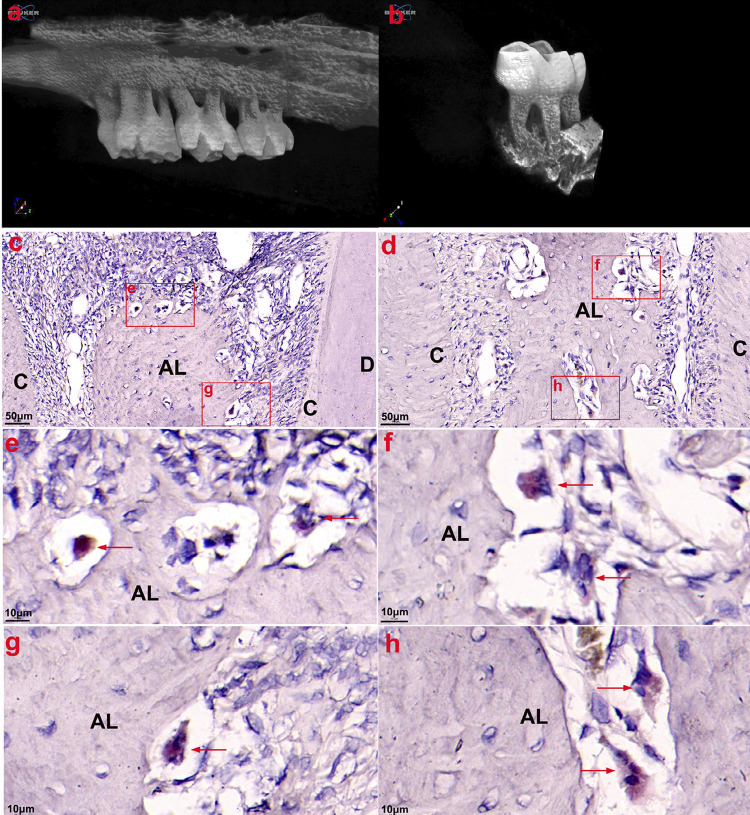
Micro-CT showed significant alveolar bone resorption of the maxillary second molar at 4 weeks after ligation. A great number of TRAP-positive multinucleated osteoclasts were observed at the edge of the upper 1/3 (c, e, and g) and middle 1/3 (d, f, and h) of the remaining alveolar bone. AL: Alveolar bone; C: Cementum; and D: Dentin

**Figure 4:**
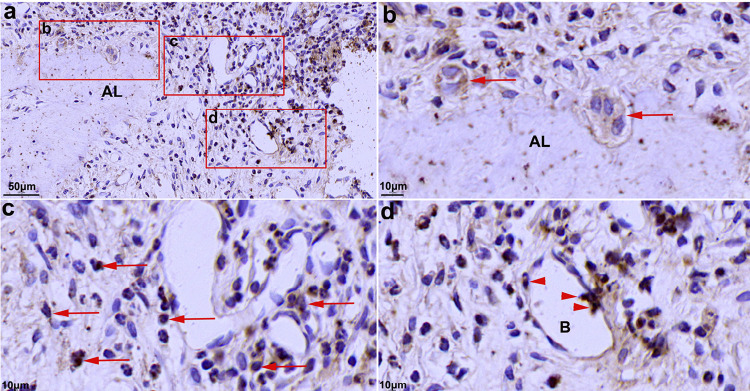
Expression of TIPE2 in rat periodontitis detected using immunohistochemistry. Multinucleated osteoclasts were TIPE2-positive (a and b). TIPE2-positive monocyte passing through the vascular wall (c). Positive expression of TIPE2 was also observed in some alveolar bone cells (d). AL: Alveolar bone; C: Cementum

**Figure 5:**
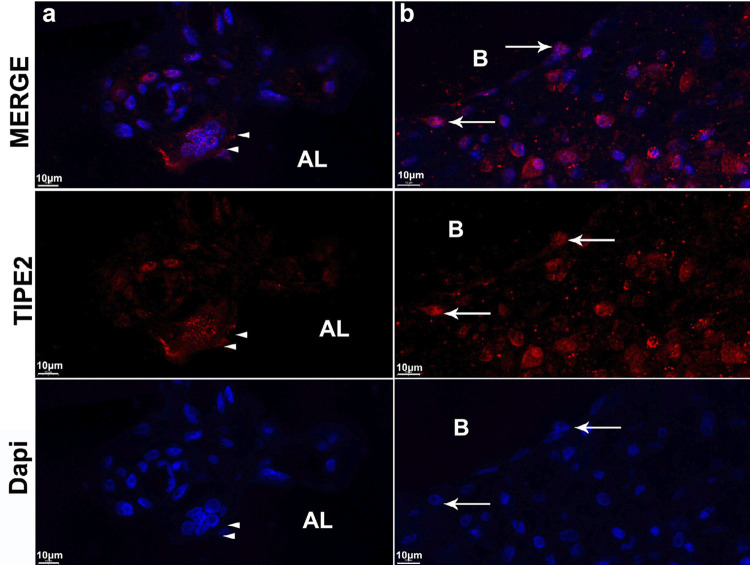
Expression of TIPE2 in rat periodontitis detected using immunofluorescence. Osteoclasts at the edge of the alveolar bone and many infiltrating cells in the periodontal infiltrating area were TIPE2-positive. TIPE2-positive monocytes passing through the vascular wall (white arrows)

Significant alveolar bone resorption of the maxillary second molar was observed at four weeks after ligation (Figure 3a and b). Numerous TRAP-positive multinucleated OCs were observed at the edge of the remaining alveolar bone (Figure 3c–h). Positive expression of TIPE2 was observed in OCs (Figures 4a, 4b, and 5a), alveolar bone cells (Figure 4d), and monocytes (Figures 4c and 5b) in periodontal infiltrating areas.

### Expression of TIPE2 in LPS-stimulated THP-1 cells *in vitro*

**Figure 6:**
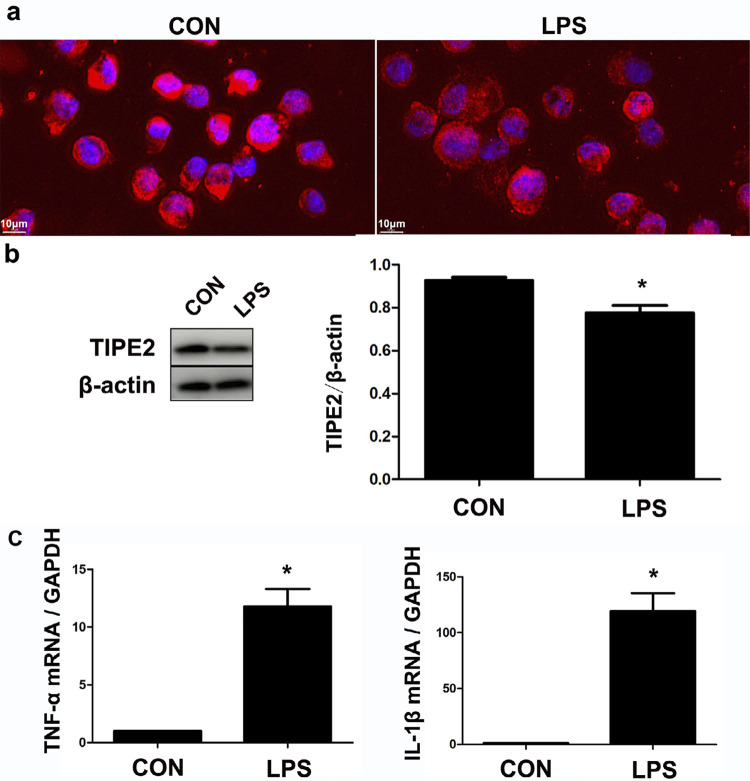
Expression of TIPE2 in THP-1cells in an inflammatory environment. Positive expression of TIPE2 was detected using immunofluorescence (a). After Pg.LPS stimulation, the TIPE2 level was decreased (b), but proinflammatory TNF-α and IL-1β were increased (c). *p<0.05

Strong positive expression of TIPE2 was detected in THP-1 cells using immunofluorescence (Figure 6a). After Pg.LPS stimulation, the levels of pro-inflammatory TNF-α and IL-1β were increased, whereas the TIPE2 level was decreased (p<0.05) (Figure 6c).

### Decreased TIPE2 promoted NF-κB p65 phosphorylation

**Figure 7:**
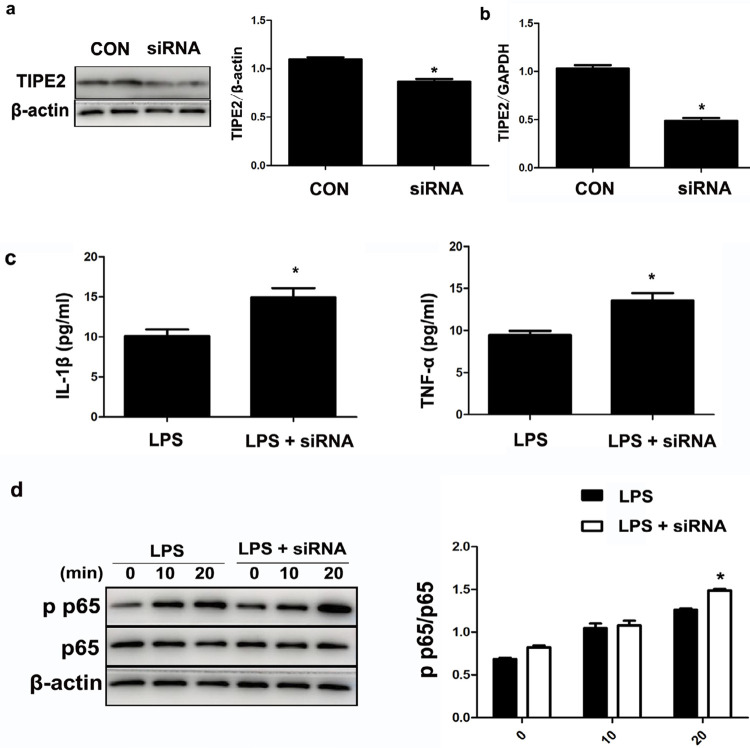
TIPE2 knockdown promoted the phosphorylation of NF-kB p65 induced by Pg.LPS. siRNA was used to knockdown the TIPE2 gene in THP-1 cells after 48 h transfection. The knockdown effect was verified using western blotting (a) and real-time PCR (b). The levels of TNFα and IL-1β were higher in the siRNA group than in the LPS group (c). After stimulation with 1 μg/ml Pg.LPS for 0, 10, and 20 min, the expression levels of p65 and pp65 were detected using western blotting (d). *p<0.05

The knockdown effect was verified using western blotting and real-time PCR. siRNA transfection suppressed TIPE2 mRNA and protein levels in the THP-1 cells (Figure 7a and b).

TIPE2 knockdown increased the levels of TNFα and IL-1β in the THP-1 cells stimulated using 0.1 μg/ml Pg.LPS for 24 h (Figure 7c). According to the results of western blotting, the expression of NF-κB p p65 was increased at 20 min in the siRNA group compared with the LPS group (Figure 7d). The results suggested that decreased TIPE2 in THP-1 cells promoted Pg.LPS-induced NF-κB p65 phosphorylation and aggravated periodontal inflammatory infiltration.

## Discussion

Periodontitis is the sixth-most prevalent disease worldwide, being caused by bacterial infection and resulting host immune response. The disease is characterized by progressive gingival inflammation and alveolar bone resorption.^16^ The severity and extent of periodontitis depend on the interaction between triggering microbial factors and the host immune system, with monocytes/macrophages playing an essential role in this process.^17,18^ The monocyte/macrophage lineage can give rise to OCs via the action of M-CSF and RANKL,^19^ which further participate in alveolar bone absorption.^20^

TIPE2 is a significant controller of the immune response in numerous conditions, including chronic hepatitis,^10^ asthma,^11^ systemic lupus erythematosus,^12^ and osteoporosis.^21^ However, the expression and function of TIPE2 in the process of periodontitis are still unclear. THP-1 cells have been extensively used to study monocyte/macrophage functions, mechanisms, and signaling pathways.^22^ In this study, the result of immunofluorescence and western blotting showed strong positive expression of TIPE2 in THP-1 cells, with the activation of the classical inflammatory signaling pathways NF-kB p65 and p38 after two weeks since ligation. Moreover, higher expression of TIPE2 was detected in the inflammatory gingival tissues. According to immunohistochemistry and immunofluorescence results, positive expression of TIPE2 was distributed in the inflammatory infiltrating cells and OCs in the marginal lacunae of the alveolar bone. Furthermore, numerous TIPE2-positive inflammatory cells were found to have entered the gingival infiltration area, which explained the increased expression of TIPE2 protein in the gingival tissues from rats with periodontitis. The results show that TIPE2 participated in the progression of rat ligation-induced periodontitis.

*Porphyromonas gingivalis* is one of the most important periodontal pathogens.^23^ Pg.LPS can induce periodontal diseases that present with severe bone loss and gingival inflammation, which is accompanied by a high number of infiltrating monocyte/macrophages.^24^ In this study, strong positive expressions of TIPE2 in THP-1 cells were decreased after Pg.LPS stimulation. It has been reported that decreased TIPE2 in peripheral blood mononuclear cells is associated with the progression of various diseases.^10-13^ We assumed that the decreased TIPE2 in the monocyte/macrophage lineage could aggravate the process of periodontitis.

To further explore the molecular mechanisms of TIPE2 in periodontitis, TIPE2 mRNA was suppressed by siRNA transfection. The results of western blotting showed that NF-κB p65 was increased at 20 min in the interference group. This result suggested that decreased TIPE2 in THP-1 cells promoted Pg.LPS-induced NF-κB p65 phosphorylation and then aggravated periodontal progressive inflammatory infiltration. Considering the essential role of TIPE2 in linking THP-1 and periodontal inflammation, interventions aimed at increasing TIPE2 may be a promising therapeutic target in periodontitis treatment by inhibiting NF-κB p65 phosphorylation.

Since TIPE2 has been identified as a critical modulator of immune response, it is imperative to comprehend its expression and function in the pathogenesis of periodontitis. This study represented the first attempt to showcase the participation of TIPE2 in the advancement of rat ligation-induced periodontitis. Furthermore, our findings indicated that reduced TIPE2 expression within the monocyte/macrophage lineage could exacerbate the progression of periodontitis. Further studies are necessary to elucidate the molecular mechanism of TIPE2 in different infiltrating immune cells of periodontitis and to develop potential adjunctive therapeutic strategies for periodontitis.^25^
